# Standard Distal Pancreatectomy Versus Radical Antegrade Modular Pancreatosplenectomy: A Systematic Review and Meta-Analysis of the Literature

**DOI:** 10.7759/cureus.105617

**Published:** 2026-03-21

**Authors:** Mark Portelli, Cressida Gauci, Khodor Kobrosliy, Jo-Etienne Abela

**Affiliations:** 1 Department of Surgery, Mater Dei Hospital, Msida, MLT

**Keywords:** distal pancreatectomy, pancreas, pancreatic cancer, ramps, tumour

## Abstract

Distal pancreatectomy is the current standard treatment for resectable distal pancreatic tumours. Radical antegrade modular pancreatosplenectomy (RAMPS) introduced a novel approach for resection to improve post-operative outcomes by optimising visualisation and vascular control through a medial-to-lateral dissection technique. This meta-analysis aims to comprehensively evaluate perioperative aspects and post-operative outcomes associated with RAMPS and standard distal pancreatectomy (SDP). A systematic literature search was conducted in PubMed, MEDLINE, Cochrane Library and Google Scholar from January 1, 2003, to August 31, 2025, using the MeSH terms 'distal pancreatectomy' and 'Radical Antegrade Modular Pancreatosplenectomy'. Eligible studies, including randomised controlled trials, cohort studies, and prospective studies, were selected for comparison using RevMan 5.3 (The Cochrane Collaboration, London, England, UK). Outcomes, such as operative time, intraoperative blood loss, length of hospital stay, lymph node yield, complications, residual margins, and recurrence, were analysed with 95% CIs, using a random-effects model. This review followed Preferred Reporting Items for Systematic Reviews and Meta-Analysis or PRISMA guidelines. Thirteen studies met the inclusion criteria involving a total of 2755 patients (1507 in the SDP group and 1248 in the RAMPS group). Our analysis revealed no statistically significant differences in length of hospital stay (mean difference (MD): 4.69 days; 95% CI: -2.30, 11.68), complications (OR: 1.21; 95% CI: 0.53, 2.77), or recurrence rates (OR: 1.47; 95% CI: 0.98, 2.23) between RAMPS and SDP. However, RAMPS demonstrated reduced operative time (MD: 60.44 minutes; 95% CI: 55.67, 65.20), intraoperative blood loss (MD: 197.13 millilitres; 95% CI: 169.56, 224.70), a higher number of harvested lymph nodes (MD: -5.91 lymph nodes; 95% CI: -8.17, -3.66), and achievement of R0 resection (OR: 0.51; 95% CI: 0.29, 0.88). This systematic review and meta-analysis demonstrates that RAMPS and SDP yield largely comparable perioperative and early oncological outcomes in patients with resectable distal pancreatic tumours. While overall complication rates, length of hospital stay, and recurrence were similar between the two techniques, RAMPS was associated with favourable intraoperative metrics, including shorter operative time, reduced blood loss, a greater lymph node yield, and higher rates of R0 resection. These findings suggest that RAMPS may offer technical and oncological advantages in selected patients; however, the current evidence is predominantly derived from non-randomised studies. Well-designed, adequately powered randomised controlled trials are required to confirm these potential benefits and to better define the role of RAMPS in the surgical management of distal pancreatic tumours.

## Introduction and background

Pancreatic cancer is an aggressive malignancy with poor survival outcomes despite advances in surgical and oncological management. Its rapid progression and high rates of local recurrence pose significant challenges in achieving curative treatment [1,2]. Surgery remains the only curative option for such lesions [3]. Resectable tumours of the pancreatic body or tail are traditionally treated via standard distal pancreatectomy (SDP). However, the aggressive nature of these tumours has raised concerns regarding positive resection margins and poor disease-free survival rates following surgery [2].

SDP is performed in a retrograde fashion with dissection commencing from the pancreatic tail towards the pancreatic neck. This left-to-right approach may result in suboptimal visualisation of posterior structures during the early stages of the operation, increasing the risk of positive posterior resection margins. In addition, vascular control is often achieved later in the procedure, which may increase the risk of intraoperative haemorrhage [1,4].

To address these limitations, radical antegrade modular pancreaticosplenectomy (RAMPS) was introduced in 2003 by Strasberg et al. [4]. This technique aims to improve oncological clearance by increasing the likelihood of achieving negative resection margins and improving lymph node yield during pancreatectomy. Unlike the conventional retrograde approach, RAMPS involves a right-to-left dissection beginning at the pancreatic neck, allowing earlier control of major vascular structures and improved visualisation of the posterior dissection plane.

Pre-operative computed tomography (CT) scans allow the hepatobiliary surgeon to assess the posterior extent of invasion of the tumour and to plan the posterior plane of dissection. Two possible approaches to dissection in RAMPS have been described in the literature: anterior RAMPS and posterior RAMPS. In the posterior approach, the adrenal gland is dissected and resected, whereas in anterior RAMPS, it is preserved [4].

Despite these theoretical advantages, the clinical benefits of RAMPS compared with SDP remain a topic of ongoing debate. This systematic review and meta-analysis, therefore, aims to compare perioperative safety and oncological outcomes of RAMPS versus SDP in patients with distal pancreatic adenocarcinoma. By incorporating the currently available evidence, this study seeks to provide a comprehensive assessment of the role of RAMPS in the surgical management of distal pancreatic tumours.

This study was previously presented as a meeting abstract at the Pancreatic Society of Great Britain and Ireland Annual Scientific Meeting 2023. The data were subsequently updated to include publications until August 2025.

## Review

Method

Search Sources and Strategy

The literature search was carried out using PubMed/MEDLINE, Cochrane Library and Google Scholar academic search engines using the ‘Advanced Search’ setting and the medical subject headings (MeSH) 'distal pancreatectomy' and 'Radical Antegrade Modular Pancreatosplenectomy'. To obtain specific studies, Boolean characters ‘OR’ and ‘AND’ were used, with wildcards such as ‘+’, “” and ‘?’. Studies published between 1 January 2003 and 31 August 2025 on the relevant topic were identified and analysed. Inclusion criteria for our meta-analysis included randomised controlled trials, non-randomised controlled trials, cohort studies and prospective studies with a follow-up duration of more than two months. The literature search was performed independently by two authors using the pre-defined MeSH terms. A third author was involved to settle any disagreements. The compiled list of results was then vetted using the research software Rayyan (Rayyan Systems Inc., Cambridge, MA, US), and all duplicates were removed from the list. From the remaining studies, qualitative studies, systematic reviews and individual case reports were excluded, narrowing the list further. Subsequently, any studies that evaluated SDP versus RAMPS were also highlighted.

*Data Extraction and Statistical Analysis*

**Figure 1:**
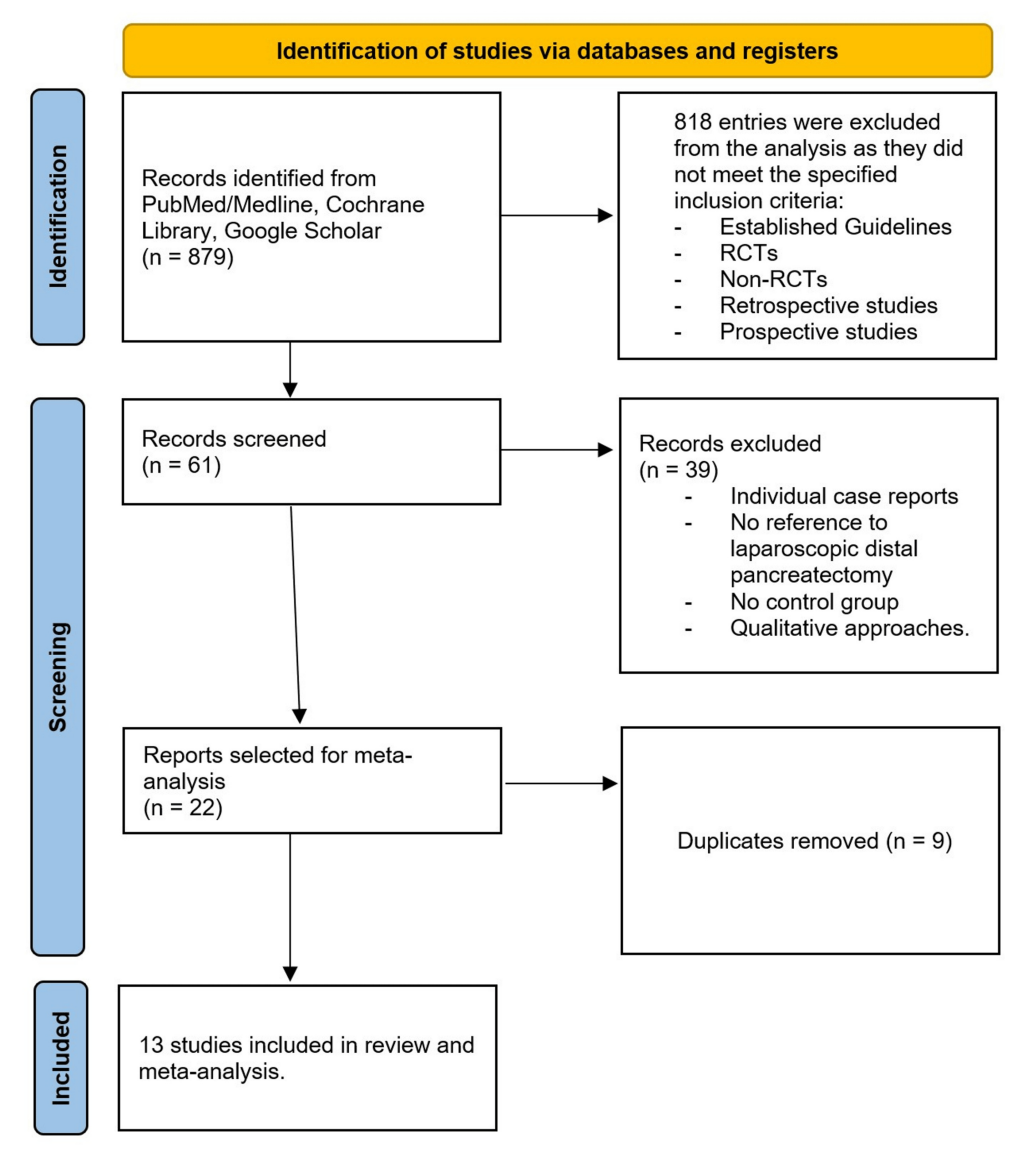
PRISMA chart of included studies PRISMA, Preferred Reporting Items for Systematic Reviews and Meta-Analysis; RCT, randomised controlled trial.

Two reviewers working independently extracted the data from the included studies. Data were compiled using Microsoft Excel (Microsoft Corp., Redmond, WA, USA), and the comparison was performed in Review Manager 5.3 (The Cochrane Collaboration, Oxford, UK) and SPSS 24.0 (IBM Corp., Armonk, NY). The initial literature search yielded 13 studies fitting the inclusion criteria and provided sufficient data for the meta-analysis. Outcomes, including operative time, intraoperative blood loss, length of hospital stay, harvested lymph nodes, complications, residual margins, and recurrence, were analysed with 95% CIs, using a random-effects model. Study heterogeneity was assessed using the I-square (I^2^) and chi-square (χ^2^) tests. Continuous outcomes were analysed using mean differences calculated as SDP minus RAMPS, with negative values indicating a benefit in favour of RAMPS. Categorical outcomes were assessed using odds ratios. Meta-analyses were performed for each outcome using the available data. A qualified statistician was involved in the design and conduct of the statistical analysis. This meta-analysis was performed following the recommendations of the Preferred Reporting Items for Systematic Reviews and Meta-Analysis or PRISMA statement and the Cochrane Collaboration statement (Figure 1) [5].

To minimise bias, the methodological quality of the included studies was critically appraised. The risk of bias in non-randomized studies-of interventions (updated guidance, version 2) was used to assess risk of bias in the 13 included non-randomised studies, and this was carried out by two separate authors [6-8]. A third author was involved to settle any disagreements.

Results

The database search yielded 879 records. After removal of duplicates and screening of titles, abstracts and subsequently full texts, 13 studies were included in the final analysis (Table 1). The literature search did not yield any randomised controlled trials on the topic. All 13 studies were retrospective cohort studies [9-21]. Ten were single-centre cohort studies [9-12,14,15,17-19,21], and three were multicentre studies [13,16,20]. Our review yielded 2755 patients undergoing distal pancreatectomy. 1507 underwent SDP, while 1248 underwent RAMPS.

**Table 1:** Summary of characteristics of included studies RAMPS, radical antegrade modular pancreatosplenectomy; SDP, standard distal pancreatectomy.

Study	Type of study	Single centre/multicentre	Participants	SDP	RAMPS	Recorded outcomes
Latorre et al. 2013 [9]	Retrospective cohort study	Single centre	25	17	8	Number of harvested lymph nodes
Park et al. 2014 [10]	Retrospective cohort study	Single centre	92	54	38	Complications, recurrence
Trottman et al. 2014 [11]	Retrospective cohort study	Single centre	26	20	6	Operative time, intraoperative blood loss, length of hospital stay, number of harvested lymph nodes, complications, clear resection margins (R0)
Abe et al. 2016 [12]	Retrospective cohort study	Single centre	93	40	53	Operative time, intraoperative blood loss, length of hospital stay, number of harvested lymph nodes, complications, clear resection margins (R0), recurrence
He et al. 2020 [13]	Retrospective cohort study	Multicentre	446	193	253	Complications, clear resection margins (R0)
Dai et al. 2021 [14]	Retrospective cohort study	Single centre	103	57	46	Operative time, intraoperative blood loss, length of hospital stay, number of harvested lymph nodes, complications
Jie et al. 2021 [15]	Retrospective cohort study	Single centre	192	114	78	Operative time, clear resection margins (R0)
Kim et al. 2021 [16]	Retrospective cohort study	Multicentre	106	53	53	Operative time, intraoperative blood loss, length of hospital stay, number of harvested lymph nodes, clear resection margins (R0), recurrence
Sutton et al. 2022 [17]	Retrospective cohort study	Single centre	353	236	117	Operative time
Li et al. 2024 [18]	Retrospective cohort study	Single centre	154	66	88	Clear resection margins (R0), recurrence
Borys et al. 2024 [19]	Retrospective cohort study	Single centre	25	13	12	Complications, clear resection margins (R0)
Kwon et al. 2024 [20]	Retrospective cohort study	Multicentre	333	130	203	Complications, clear resection margins (R0), recurrence
Yin et al. 2025 [21]	Retrospective cohort study	Single centre	581	384	197	Complications, clear resection margins (R0), recurrence

The objectives were addressed by performing seven meta-analyses, one for each of the seven predefined datasets. For each meta-analysis, we used all available data corresponding to the specific parameter under investigation. Where available, means and standard deviations were preferred.

Operative Time

**Figure 2:**
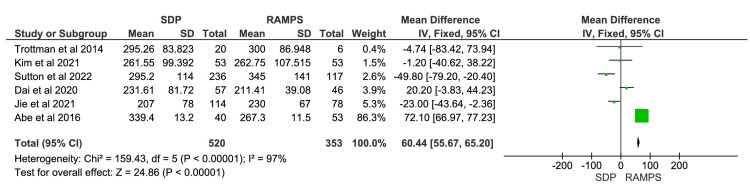
Meta-analysis of operative time IV, intravenous; RAMPS, radical antegrade modular pancreatosplenectomy; SDP, standard distal pancreatectomy. [11,12,14-17]

Operative time was defined as the time from initial incision to final suture. A total of six studies were included in the meta-analysis, involving 873 participants: 520 in the SDP group and 353 in the RAMPS group [11,12,14-17]. The meta-analysis showed a significantly shorter operative time in the RAMPS group (Figure 2). The mean difference in operative time between SDP and RAMPS was 60.44 minutes, with a 95% CI of 55.67 to 65.20. The heterogeneity values were χ^2^=159.43 (p<0.00001) and I^2^=97%.

Intraoperative Blood Loss

**Figure 3:**
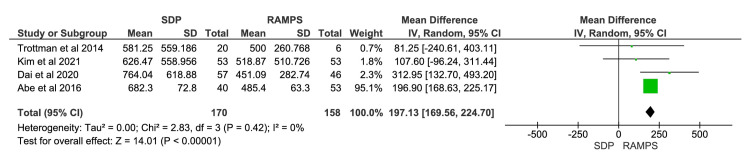
Meta-analysis of intraoperative blood loss IV, intravenous; RAMPS, radical antegrade modular pancreatosplenectomy; SDP, standard distal pancreatectomy. [11,12,14,16]

A total of four studies were included in the meta-analysis, involving 328 participants: 170 in the SDP group and 158 in the RAMPS group [11,12,14,16]. The meta-analysis showed a significantly lower intraoperative blood loss in the RAMPS group (Figure 3). The mean difference in intraoperative blood loss between SDP and RAMPS was 197.13 millilitres, with a 95% CI of 169.56 to 224.70. The heterogeneity values were χ^2^=2.83 (p<0.00001) and I^2^=0%.

Length of Hospital Stay

**Figure 4:**
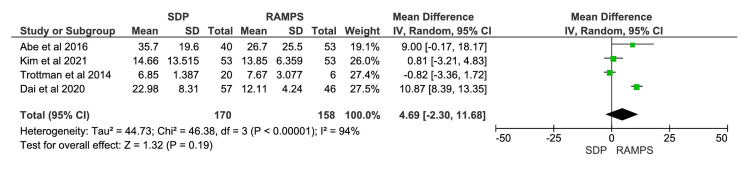
Meta-analysis of duration of hospital stay IV, intravenous; RAMPS, radical antegrade modular pancreatosplenectomy; SDP, standard distal pancreatectomy. [11,12,14,16]

A total of four studies were included in the meta-analysis, involving 328 participants: 170 in the SDP group and 158 in the RAMPS group [11,12,14,16]. The mean difference in length of hospital stay between SDP and RAMPS was 4.69 days, with a 95% CI of -2.30 to 11.68. The results were not statistically significant, since the CI included zero. The heterogeneity values were χ^2^=46.38 (p<0.00001) and I^2^=94% (Figure 4).

Number of Harvested Lymph Nodes

**Figure 5:**
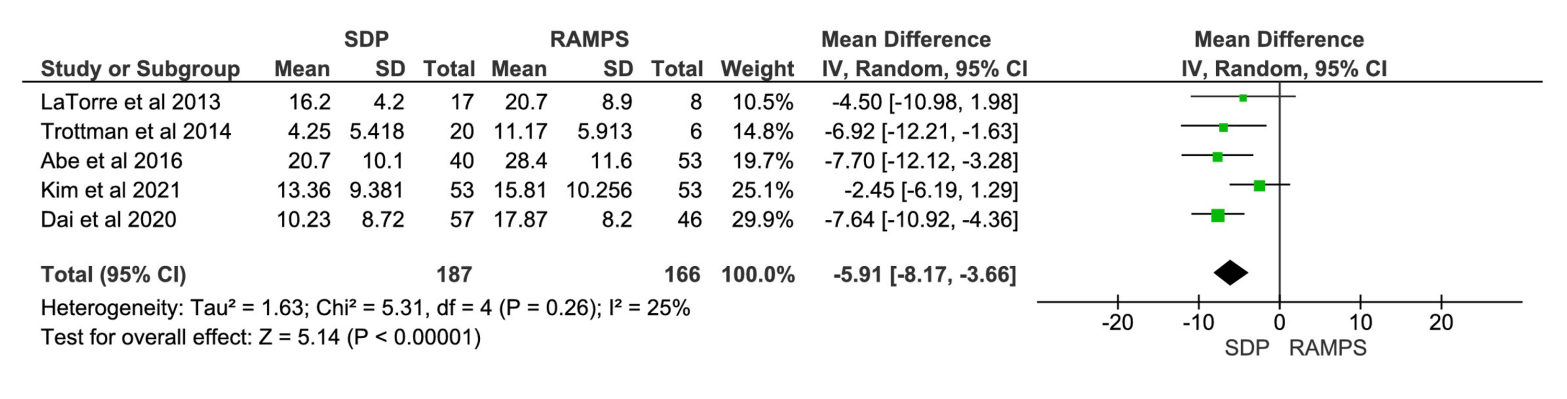
Meta-analysis of harvested lymph node count IV, intravenous; RAMPS, radical antegrade modular pancreatosplenectomy; SDP, standard distal pancreatectomy. [9,11,12,14,16]

A total of five studies were included in the meta-analysis, involving 353 participants: 187 in the SDP group and 166 in the RAMPS group [9,11,12,14,16]. The meta-analysis demonstrated that the number of harvested lymph nodes was significantly higher in the RAMPS group (Figure 5). The mean difference in the number of harvested lymph nodes between SDP and RAMPS was -5.91, with a 95% CI of -8.17 to -3.66. The heterogeneity values were χ^2^=5.31 (p=0.26) and I^2^=25%.

Complications

**Figure 6:**
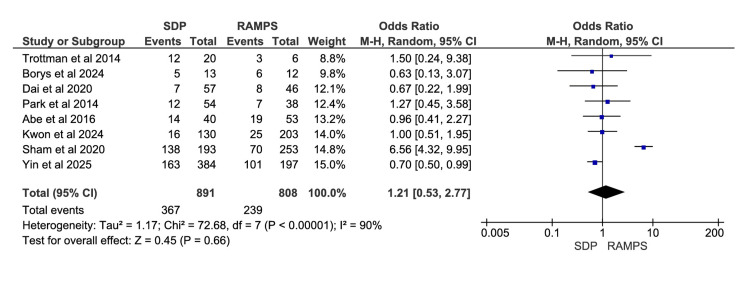
Meta-analysis of postoperative complications RAMPS, radical antegrade modular pancreatosplenectomy; SDP, standard distal pancreatectomy. [10-14,19-21]

A total of eight studies were included in the meta-analysis, involving 1699 participants: 891 in the SDP group and 808 in the RAMPS group [10-14,19-21]. The odds ratio for the frequency of complications in SDP versus RAMPS was 1.21, with a 95% CI of 0.53 to 2.77. The results were not statistically significant, since the CI included one. The heterogeneity values were χ^2^=72.68 (p<0.00001) and I^2^=90% (Figure 6).

Clear Resection Margins (R0)

**Figure 7:**
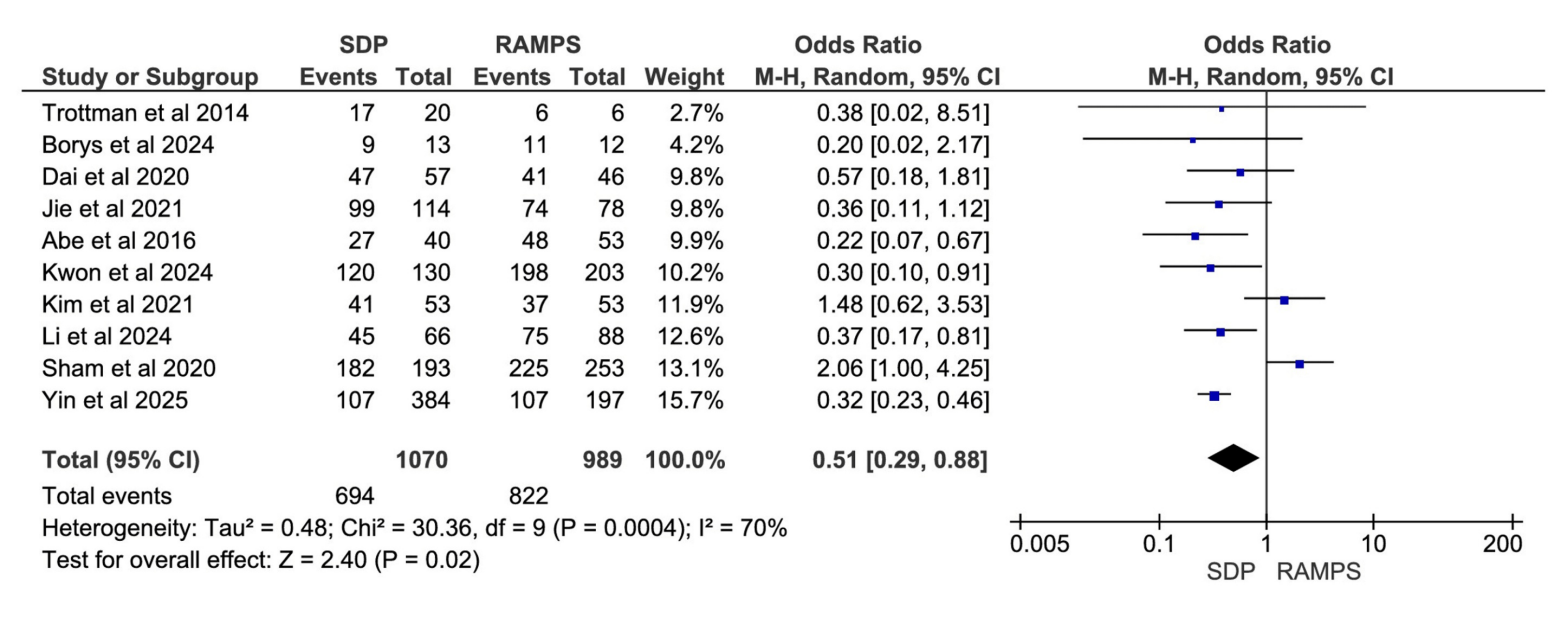
Meta-analysis of achievement of R0 resection RAMPS, radical antegrade modular pancreatosplenectomy; SDP, standard distal pancreatectomy. [11-16,18-21]

A total of ten studies were included in the meta-analysis, involving 2059 participants: 1070 in the SDP group and 989 in the RAMPS group [11-16,18-21]. The frequency of clear resection margins (R0) significantly favoured RAMPS, with a pooled odds ratio of 0.51 and a 95% CI of 0.29 to 0.88. The heterogeneity values were χ^2^=30.36 (p<0.0004) and I^2^=70% (Figure 7).

Recurrence

**Figure 8:**
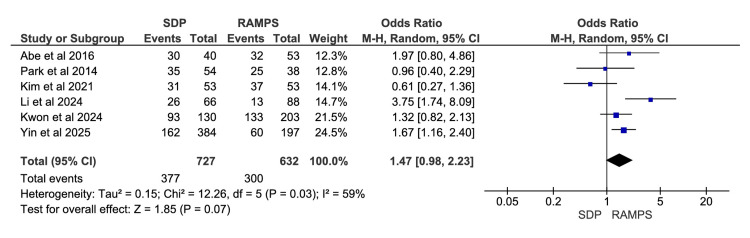
Meta-analysis of recurrence rates RAMPS, radical antegrade modular pancreatosplenectomy; SDP, standard distal pancreatectomy. [10,12,16,18,20,21]

A total of six studies were included in the meta-analysis, involving 1359 participants: 727 in the SDP group and 632 in the RAMPS group [10,12,16,18,20,21]. The odds ratio for the frequency of recurrence in SDP versus RAMPS was 1.47, with a 95% CI of 0.98 and 2.23. The results were not statistically significant, since the CI included one. The heterogeneity values were χ^2^=12.26 (p=0.03) and I^2^=59% (Figure 8).

Risk of Bias Assessment

**Figure 9:**
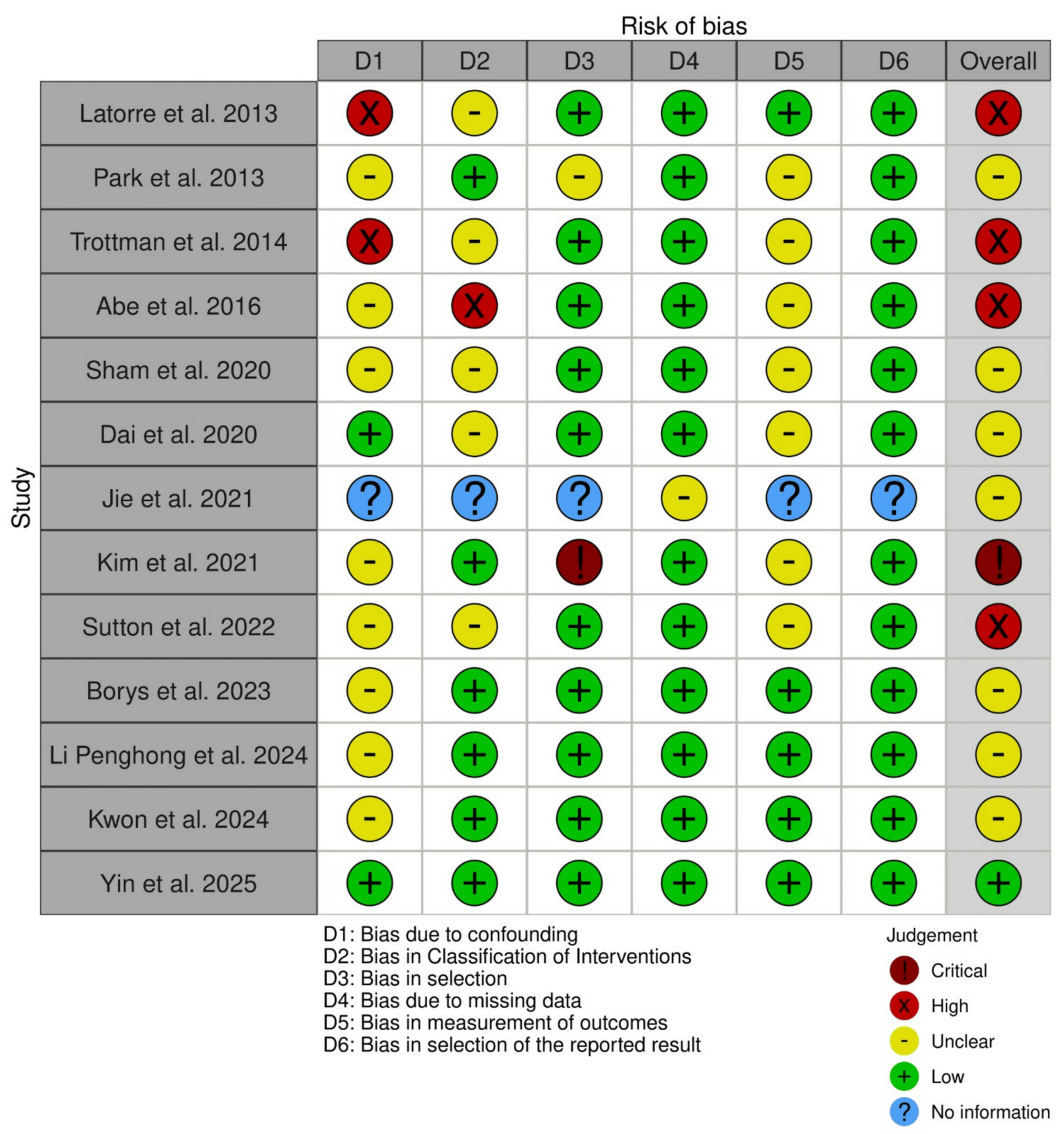
Risk of bias assessment traffic-light plot using the ROBINS-I tool ROBINS-I, risk of bias in non-randomized studies-of interventions. [9-21]

The risk of bias across the included studies was moderate to high. Jie et al. could not be fully assessed with regard to risk of bias, since this was published as an abstract; sufficient data regarding methodology were not included [15]. Kim et al. were deemed to have an overall critical risk of bias due to the researchers’ method of participant selection, which reduced the reader’s confidence in the results related to length of hospital stay, R0 resection and recurrence [16]. This also limits the generalisability of the findings, as patients with more advanced disease were excluded, and therefore the results may not be directly applicable to standard clinical practice. Four studies were deemed to have a serious risk of bias, mainly owing to issues related to confounding and classification of the implemented interventions [9,11,12,17]. Seven of the remaining studies were deemed to have a moderate risk of bias [10,13-15,18,19,20]. One study by Yin et al. had a low risk of bias [21]. A summary of the risk of bias assessment for the included studies is shown in Figure 9.

Discussion

Pancreatic adenocarcinoma of the body and tail is characterised by an aggressive biological course and high rates of local recurrence and metastasis, making curative surgical resection challenging [19]. Despite significant advances in modern medical and surgical practices, surgery remains the cornerstone of treatment for left-sided pancreatic tumours. However, achieving optimal oncological clearance while minimising perioperative morbidity remains challenging.

SDP has historically been associated with risks of haemorrhage and difficulty in clearing posterior resection margins. These challenges led to the development of RAMPS by Strasberg in 2003 to improve post-operative outcomes by optimising visualisation and vascular control through a right-to-left dissection approach [4]. The posterior dissection plane in RAMPS can be adjusted either anterior or posterior to the left adrenal gland, based on the depth of tumour infiltration [1,18].

Strasberg et al. initially described the RAMPS technique, based on an improved understanding of pancreatic lymphatic drainage, with the primary aim of increasing the rate of margin-negative (R0) resection in patients undergoing distal pancreatectomy [4]. Although several early comparative studies failed to demonstrate a statistically significant improvement in complete resection rates with RAMPS when compared with SDP, the pooled findings of our meta-analysis suggest a relative advantage of RAMPS in achieving clear resection margins [10,12,13].

This apparent discrepancy between individual studies and the pooled analysis may be explained by several factors. First, considerable heterogeneity exists in pathological assessment techniques across institutions, particularly regarding specimen orientation, inking of margins, and definitions of what constitutes an R0 resection. Such variability may lead to inconsistent reporting of margin status and potentially obscure true differences between surgical approaches. Second, variation in surgeon experience and institutional volume may also influence oncological outcomes, as RAMPS is a technically demanding procedure that requires familiarity with complex retroperitoneal anatomy.

The observed favourability towards RAMPS in achieving R0 resection in our analysis suggests that, when performed in appropriate settings, the technique can deliver the oncological benefits originally proposed by Strasberg and colleagues. This supports the concept that the systematic right-to-left dissection employed in RAMPS facilitates improved exposure of critical vascular structures and posterior planes, thereby enhancing the likelihood of achieving negative margins. Furthermore, the statistically significant increase in the number of harvested lymph nodes observed in the RAMPS group further reinforces the anatomical and oncological rationale underpinning this approach. Taken together, these findings indicate that RAMPS appears to offer technical and pathological advantages over SDP, particularly in centres with adequate expertise, while highlighting the importance of standardised pathological reporting and surgical technique when interpreting oncological outcomes.

In addition to the oncological parameters, RAMPS demonstrated significant perioperative advantages, including a statistically significant reduction in operative time and a reduction in intraoperative blood loss. These findings suggest that early vascular control and structured dissection may streamline the surgical process, possibly leading to immediate perioperative benefits in patients with reduced intraoperative risk. Reduced blood loss is particularly relevant in pancreatic surgery, where transfusion requirements have been associated with increased postoperative morbidity [22].

Despite these advantages, our meta-analysis demonstrated no statistically significant differences between RAMPS and SDP in length of hospital stay, post-operative complications or recurrence rates. While trends favour RAMPS for recurrence, these did not reach statistical significance, likely reflecting limited power, heterogenous follow-up duration and variation in adjuvant treatment strategies. These findings suggest that while RAMPS may offer intraoperative and oncological benefits, these do not necessarily translate into demonstrable differences in short-term post-operative outcomes or recurrence within the available follow-up periods. In terms of recurrence, interpretation is limited by heterogeneity in follow-up duration and adjuvant therapy reporting across studies.

Comparison with previous meta-analyses highlights both similarities and differences. Consistent with Huo et al., our analysis demonstrated reduced intraoperative blood loss with RAMPS [23]. In contrast to Jiang et al., who reported shorter operative time with SDP and no difference in R0 resection, our analysis favoured RAMPS for operative time, lymph node harvest and R0 resection [1]. These discrepancies likely reflect differences in study inclusion, patient selection, surgical expertise and the evolving adoption of RAMPS over time.

The overall quality of the available evidence is limited by the retrospective nature of the included studies, an overall moderate risk of bias and heterogeneity across outcomes. A key limitation of this meta-analysis is the absence of randomised controlled trials comparing RAMPS with SDP. To date, no randomised controlled trial on this topic has been published. All included studies were retrospective cohort studies, introducing the potential for selection bias, confounding and institutional variability in surgical practice. Patients selected for RAMPS may differ in tumour characteristics, surgeon expertise or institutional volume, which may influence perioperative and oncological outcomes.

To ensure a complete review of the literature, we included heterogeneous data when possible, in order to ensure generalisability of the results. Nevertheless, multiple data sets from separate studies needed to be excluded from the final statistical analysis for statistical reasons. Definitive conclusions regarding survival benefit require high-quality prospective studies. The ongoing Chinese Study Group for Pancreatic Cancer-3 (CSPAC-3) trial, a multicenter prospective phase III randomised control trial comparing RAMPS with SDP for resectable pancreatic ductal adenocarcinoma of the body and tail, is expected to provide critical insight into long-term oncological outcomes [24].

## Conclusions

In conclusion, this meta-analysis suggests that RAMPS is associated with significant perioperative advantages, including reduced operative time and intraoperative blood loss, as well as improved pathological outcomes in terms of lymph node yield and R0 resection. Post-operative morbidity, length of hospital stay, and recurrence rates appear comparable between RAMPS and SDP. At present, the selection of surgical methods remains subject to surgeon preference and experience, thus introducing an element of variability that complicates the evaluation of outcome measures from the procedure.

Given the observational nature of the available evidence and the overall moderate risk of bias across included studies, these findings should be interpreted with caution. While RAMPS shows promising technical and oncological features, definitive conclusions regarding long-term overall oncological superiority cannot yet be established. Further high-quality, high-powered, multicenter, randomised controlled trials, such as the ongoing CSPAC-3 trial, are required to validate the long-term efficacy and safety of the RAMPS procedure approach. Standardisation of operative technique, pathological assessment, and surgeon training may further enhance the reproducibility and evaluation of RAMPS in clinical practice.
