# Correction: Standard Distal Pancreatectomy Versus Radical Antegrade Modular Pancreatosplenectomy: A Systematic Review and Meta-Analysis of the Literature

**DOI:** 10.7759/cureus.c421

**Published:** 2026-04-03

**Authors:** Mark Portelli, Cressida Gauci, Khodor Kobrosliy, Jo-Etienne Abela

**Affiliations:** 1 Department of Surgery, Mater Dei Hospital, Msida, MLT

This article has been corrected as follows: the author order was incorrectly entered at the time of submission and did not accurately reflect the intended order of contributions. The article has been updated to display the intended author order.

